# Prognostic Value of Arm Circumference for Cardiac Damage and Major Adverse Cardiovascular Events: A Friend or a Foe? A 2-Year Follow-Up in the Northern Shanghai Study

**DOI:** 10.3389/fcvm.2022.816011

**Published:** 2022-06-23

**Authors:** Yixing Zheng, Ji Zhang, Zhongyuan Ren, Weilun Meng, Jiamin Tang, Song Zhao, Chen Chi, Jing Xiong, Jiadela Teliewubai, Rusitanmujiang Maimaitiaili, Yawei Xu, Yi Zhang

**Affiliations:** Department of Cardiology, Shanghai Tenth People’s Hospital, Tongji University School of Medicine, Shanghai, China

**Keywords:** cardiovascular, anthropometric measurement, target organ damage, health care, elderly, Chinese

## Abstract

**Background:**

The high prevalence of cardiovascular diseases globally causes a great social burden and much individual suffering. The effective recognition of high-risk subjects is critical for primary prevention in the general population. In the elderly cohort, anthropometric measurements may have different prognostic values. Our study aimed to find convincing anthropometric measures to supplement conventional risk factors for major adverse cardiovascular events (MACEs) in the elderly cohort.

**Materials and Methods:**

A total of 1,576 elderly participants (44.5% male, aged 72.0 ± 6.0 years) recruited into the Northern Shanghai Study (2014–2015) were followed up between 2016 and 2017. Following the standard guideline for cardiovascular risk evaluation, all conventional cardiovascular risk factors were assessed. The body measures were made up of body weight, body height, hip circumference, waist circumference, and middle-upper arm circumference (MUAC). Organ damage (OD) markers for cardiac, vascular, and renal diseases will be evaluated by the standardized methods.

**Results:**

After the average 571 (±135) days of follow-up, a total of 90 MACEs (5.7%) occurred, i.e., 13 non-fatal myocardial infarction, 68 non-fatal stroke, and 9 cardiovascular deaths. Univariable COX survival analysis revealed that only MUAC could validly predict MACEs among anthropometric characters [adjusted hazard ratio (HR) 0.89; 95% confidence interval (CI) 0.82–0.96]. In Kaplan-Meier analysis, the group of high MUAC showed the lowest MACE risk (log-rank *p* = 0.01). Based on OD analysis, MUAC was independently linked to higher risk of left ventricular hypertrophy (LVH) in women and left ventricular diastolic dysfunction (LVDD) in both men and women. In adjusted COX analysis, only MUAC indicated statistical significance, but all other anthropometric parameters such as BMI, waist circumference, and waist-to-hip ratio (WHR) did not indicate significance. The higher level of MUAC remained a protective factor in fully adjusted models (HR: 0.73; 95% CI: 0.59–0.91), with *p*-values markedly significant in men (HR: 0.69; 95% CI: 0.49–0.97) and marginally significant in women (HR: 0.0.77; 95% CI: 0.59–1.01). After considering all factors (i.e., cardiovascular risk factors, MUAC, BMI, and WHR), the fully adjusted COX regression analysis demonstrated that the increased MUAC level was linked to decreased MACE risk in both men (HR: 0.57; 95% CI: 0.37–0.88) and women (aHR: 0.64; 95% CI: 0.46–0.93).

**Conclusion:**

Despite being associated with a higher rate of cardiac damage, higher MUAC independently and significantly conferred protection against the MACE, in the elderly cohort.

## Introduction

Cardiovascular diseases (CVDs) remain the leading cause of disease burden in the world. Cases of CVDs dramatically increased to 525 million worldwide in 2019, almost twice the number in 1990. Meanwhile, years lived with disability (YLDs) caused by CVDs have also doubled over 30 years (1), from 17.7 to 34.4 million. The occurrence of the major adverse cardiovascular events (MACE), including non-fatal myocardial infarction, non-fatal stroke, and cardiovascular death indicates the most severe aspects of CVDs. Thereinto, ischemic heart disease and cerebrovascular disease (stroke) contribute to 85.1% of all CVD deaths. Furthermore, they are globally increasing (2) and become the leading causes of total years of life lost (YLLs).

The prevention of the MACE starts from the control of risk factors of CVD. According to the insights obtained from the Framingham Heart Study, the general CVD risk profile in primary care included age, hyperlipidemia, smoking and diabetes mellitus (DM), and hypertension (3). Beyond that, chronic kidney disease (CKD) could also be regarded as a predictor of CVDs after adjustment for conventional CVD risk factors (4).

Obesity classified by weight has been recognized as an independent predictor of CVDs since 30 years ago (5). Further studies use a BMI over 30 kg/m^2^ as the definition of obesity, which is defined as a BMI over 28 kg/m^2^ in Chinese, correcting for the racial difference. However, the meta-analysis conducted by Flegal et al. (6) revealed that overweight (BMI of 25 to < 30) and grade 1 obesity (BMI of 30 to <35) were associated with lower all-cause mortality, in comparison with normal weight. A similar phenomenon was observed in studies in the field of CVDs and has been referred to as obesity paradox (7–9).

Body mass index cannot veritably reflect the nutritional status of the elderly cohort, because body height can be interfered by spinal deformities and body weight can be disturbed by fluid retention. Moreover, the BMI neglects the body composition and the distribution of different components. There are other anthropometric measures that can immediately provide this information without X-ray or MRI, such as waist circumference (WC), waist-to-hip ratio (WHR), and middle-upper arm circumference (MUAC). MUAC is a promising but long-ignored parameter. The cross-section of middle-upper arm is composed of humerus, upper arm muscles, subcutaneous fat, and skin which are fat-free-mass and peripheral fat. Both fat-free-mass and peripheral fat are favorable body components, compared with trunk fat (10). A previous study confirmed that higher MUAC was linked to lower all-cause mortality among the general population in the United States (11). In elderly patients with CVDs, MUAC was proved to serve as an independent predictor of survival (12).

The primary aim of our study was to examine and compare the associations of anthropometric parameters, including BMI, WC, WHR, and MUAC with MACEs among the community-dwelling elderly cohort. In addition, by analyzing the relationship between anthropometric parameters and organ damage (OD), we aimed to find out whether OD plays an intermediate role between anthropometric parameters and MACEs.

## Patients and Methods

### Study Population

The Northern Shanghai Study (13) (NSS, ClinicalTrials.gov Identifier: NCT02368938) is an ongoing prospective population study based on community-dwelling elderly citizens in the northern Shanghai. The preliminary sample size is expected to be 4,000 participants. From July 2014 to August 2015, 1,721 subjects were invited, of whom 1,599 participants (92.9%) were enrolled in the NSS. A total of 1,576 participants (98.6%) completed the 2-year follow-up. To start the study, participants must meet the following criteria (1): age ≥ 65 years old; (2) local residents of urban communities in Northern Shanghai; and (3) highly possibility to accept the follow-up in the next decade. The exclusion standards were as follows: (1) suffering from severe heart failure (NYHA IV) or end-stage renal dysfunction (CKD ≥ 4 stage), (2) diagnosed cancer or other severe diseases suggesting life expectancy less than 5 years, and (3) history of either hemorrhage stroke, ischemia stroke, or any other type of stroke within 3 months.

The Ethics Committee of Shanghai Tenth People’s Hospital has already approved the Northern Shanghai Study. Informed consent was obtained from all participants in written form.

### Social, Clinical, Biological, and Anthropometric Parameter Assessment

All participants enrolled in this study were investigated with the prepared standard questionnaire, including basic information such as gender, age, and family history of CVD, as well as the history and current status of smoking. The previous diagnostics of DM, hypertension, coronary heart disease (CHD), stroke, and type of stroke was collected during the interview as well.

All participants were asked to fast overnight by community workers on the previous day. Qualified nurses then obtained venous blood samples by phlebotomizing and collected urine samples. Department of Laboratory Medicine of Shanghai Tenth People’s Hospital conducted all sample tests and reported back the results of biological marker detection including serum lipid, Fasting Plasma Glucose (FPG, measured by glucose oxidase method), and serum creatinine (Scr). The Estimated Glomerular Filtration Rate (eGFR) was calculated by the Asian-modified CKD-EPI method (14).

The calibrated electronic scale was used to measure body weight in 0.1 kg precision while tape-measure was utilized for body height. The definition of obesity was BMI ≥ 28 kg/m^2^ in our study. Hip circumference was determined according to the distance around the greatest protrusion of the buttocks. To measure central obesity, WC was used as the midpoint between the iliac crest and the last rib. In our study, the definition of central obesity was WC ≥ 85 cm for men and ≥ 80 cm for women (15), respectively. Besides, MUAC, the circumference of the left upper arm, was measured at the midpoint between the shoulder and the elbow without cloths at room temperature. The midpoint was located with a soft tape at the halfway between the tip of the acromial process and the tip of the olecranon process. The examiner would mark the midpoint on participants’ skin. Then, the participants were asked to extend their arm alongside the trunk. The measure of MUAC should be gentle to avoid tissue compression. Body height, hip circumference, WC, and MUAC were all individually measured and recorded to the nearest 0.1 cm.

### Organ Damage

In our study, cardiac damage included left ventricular hypertrophy (LVH) and left ventricular diastolic dysfunction (LVDD). Vascular damage was composed of carotid intima-media thickness (IMT) >0.9 mm, carotid-femoral pulse wave velocity (CF-PWV) >12 m/s, and ultrasound-detected carotid plaque. Echocardiography and carotid ultrasonography were performed by a single proficient cardiologist independent of previous results. The equipment utilized in all measurements was a MyLab 30 CV machine (ESAOTE SpA, Genoa, Italy), and the measuring protocol was performed according to the American Society of Echocardiography (ASE) recommendations. Renal damage was identified as eGFR < 60 ml/min/1.73 m^2^. More details about the measurement and calculation procedures could be referred to in previously published articles (13, 16).

### Follow-Up

All participants were followed up by telephone interview or field interview, for up to a maximum of 2 years after their enrollment. During this period, the occurrence of newly diagnosed diseases was also tracked by self-report, including non-fatal myocardial infarction, non-fatal stroke, along with death, and cause of death. The exact event time was then recorded. In our study, the MACE includes non-fatal myocardial infarction, non-fatal stroke, and cardiovascular death.

### Statistical Analysis

Male and female participants were divided into three groups according to the tertiles of the MUAC level (low group: male MUAC ≤ 26.3 cm, female MUAC ≤ 25.9 cm; mid group: male 26.3 < MUAC ≤ 28.5 cm, female 25.9 < MUAC ≤ 28.0 cm; and high group: male MUAC > 28.5 cm, female > 28.0 cm).

Univariable analysis was applied to every conventional risk factor and the nominated risk factor. Kaplan-Meier analysis was applied to obesity, central obesity, and level-stratified MUAC. Univariable logistic regression was applied to, respectively, investigate the association between anthropometric factors and organ damage (OD). Furthermore, multivariate stepwise logistic regression models, with anthropometric factors and conventional risk factors together, were built to compare different anthropometric factors in association with OD. The respective application of Cox proportional hazard models to anthropometric factors, including MUAC, BMI, WC, and WHR, aimed to estimate the adjusted hazard ratios (aHR) for the MACE, with 95% confidence intervals (CIs) being calculated. The Cox regression models were adjusted at different levels. The final model involved MUAC, BMI, and WC in a unitive model with other risk factors including age, gender, hypertension, DM, hyperlipidemia, smoking, eGFR, and stroke history, along with CHD family history.

All continuous variables were described using mean ± standard deviation (SD). Categorical variables were presented *via* frequencies (absolute number) together with proportion (percentage) in parenthesis. Student’s *t*-test, Analysis of variance (ANOVA), and χ^2^-test were utilized to compare continuous variables in two groups, continuous variables in three groups, and categorical variables, respectively. Statistical analyses were performed with SPSS (version 18, SPSS Inc., Chicago, IL, United States). The *p*-value < 0.05 was considered statistically significant.

## Results

### Baseline Characteristics

The baseline characteristics of all participants grouped by gender are shown in Table 1, including conventional CVD risk factors, diseases, and anthropometric parameters. A total of 1,576 participants were composed of 701 men (44.5%) and 875 women (55.5%).

**TABLE 1:** Baseline characteristics.

Characteristic	Overall	Male	Female	*P*-value
n, (%)	1,576	701 (44.5)	875 (55.5)	
**Cardiovascular risk factors**				
Age (years)	72.0 ± 6.0	72.0 ± 5.9	71.9 ± 6.1	0.84
Family history of premature CVD, n (%)	321 (20.4)	123 (17.5)	198 (22.6)	0.01*
Smoking, n (%)	212 (13.5)	202 (28.8)	10 (1.1)	<0.01*
TC (mmol/L)	5.22 ± 1.01	4.93 ± 0.99	5.45 ± 0.97	<0.01*
TG (mmol/L)	1.61 ± 0.93	1.54 ± 0.85	1.66 ± 0.99	0.02*
HDLC (mmol/L)	1.38 ± 0.36	1.28 ± 0.33	1.46 ± 0.36	<0.01*
LDLC (mmol/L)	3.20 ± 0.85	3.04 ± 0.84	3.33 ± 0.83	<0.01*
SBP (mmHg)	134.6 ± 17.7	134.7 ± 16.8	134.6 ± 18.4	0.91
DBP (mmHg)	79.2 ± 9.2	80.3 ± 9.3	78.3 ± 9.0	<0.01*
eGFR ml/m in/1.73 m^2^)	82.9 ± 15.2	82.3 ± 15.3	83.3 ± 15.1	0.17
**Anthropometric character**				
Height (cm)	159.9 ± 8.2	166.4 ± 6.0	154.7 ± 5.7	<0.01*
Weight (Kg)	62.4 ± 10.6	67.6 ± 10.2	58.3 ± 8.9	<0.01*
Waist circumference (cm)	85.8 ± 9.7	87.8 ± 9.8	84.1 ± 9.4	<0.01*
Hip circumference (cm)	97.1 ± 7.2	97.3 ± 6.8	97.0 ± 7.5	0.50
MUAC (cm)	27.2 ± 2.7	27.5 ± 2.6	27.0 ± 2.7	<0.01*
BMI (kg/m^2^)	24.4 ± 3.5	24.4 ± 3.3	24.4 ± 3.6	0.995
WHR	0.88 ± 0.06	0.90 ± 0.06	0.87 ± 0.06	<0.01*
Obesity, n (%)	228 (14.5)	94 (13.4)	134 (15.3)	0.29
Central obesity, n (%)	1,032 (65.5)	433 (61.8)	599 (68.5)	0.01*
**Diseases**				
DM, n (%)	347 (22.0)	151 (21.5)	196 (22.4)	0.68
Hypertension, n (%)	1,014 (64.3)	461 (65.8)	553 (63.2)	0.29
Hyperlipidemia, n (%)	1,051 (66.7)	427 (60.9)	624 (71.3)	<0.01*
Stroke history, n (%)	315 (20.0)	128 (18.3)	187 (21.4)	0.13
CHD history, n (%)	540 (34.3)	229 (32.7)	311 (35.5)	0.23
**Target organ damage**				
LVH, n (%)	192 (12.2)	77 (11.0)	115 (13.2)	0.19
LVDD, n (%)	206 (13.4)	59 (8.8)	147 (17.1)	<0.01*
IMT > 0.9 mm, n (%)	66 (4.2)	41 (5.9)	25 (2.9)	<0.01*
CF-PWV > 12 m/s, n (%)	197 (12.9)	82 (12.2)	115 (13.5)	0.48
Carotid plaque, n (%)	1,072 (68.0)	484 (68.9)	588 (67.3)	0.48
Renal damage, n (%)	132 (8.4)	60 (8.5)	72 (8.2)	0.83
**Follow-up**				
MACE, n (%)	90 (5.7)	36 (5.1)	54 (6.2)	0.38
Non-fatal myocardial infarction, n (%)	13 (0.8)	10 (1.4)	3 (0.3)	0.02*
Non-fatal stroke, n (%)	68 (4.3)	22 (3.1)	46 (5.3)	0.04*
Cardiovascular death, n (%)	9 (0.6)	4 (0.6)	5 (0.6)	1.00

*Continuous data are presented as mean ± standard deviation and categorical variables by absolute numbers or percentage. Continuous variables and categorical variables were compared by Student’s t-test and χ^2^ test, respectively.*

*P-values < 0.05 are considered significant.

*CVD, cardiovascular disease; TC, triglyceride; TG, total cholesterol; HDLC, high density lipoprotein cholesterol; LDLC, low density lipoprotein cholesterol; SBP, systolic blood pressure; DBP, diastolic blood pressure; eGFR, estimated glomerular filtration rate; MUAC, middle-up arm circumference; BMI, body mass index; WHR, waist-to-hip ratio; DM, diabetes mellitus; CHD, coronary heart disease; MACE, major adverse cardiovascular event.*

Of the total participants, 202 (28.8%) male participants were current smokers, while only 10 (1.1%) female participants were current smokers (*p* < 0.01). Female participants reported observably higher levels than male participants in terms of all blood lipid indices and diastolic blood pressure (DBP) levels. Besides, the number of women with family history of premature CVDs was significantly larger than that of men.

In anthropometric characters, male participants were generally taller and heavier than female participants. Therefore, there existed no statistical difference in the BMI and obesity between men and women (BMI 24.4 ± 3.3 vs. 24.4 ± 3.6, *p* = 0.995). In general, men have relatively wider WCs (87.8 ± 9.8 vs. 84.1 ± 9.4, *p* < 0.01). Considering different diagnostic criterions, however, women had a higher rate of central obesity (433, 61.8% vs. 599, 68.5%, *p* = 0.01). In addition, women had thicker arms than men (MUAC: 27.5 ± 2.6 vs. 27.0 ± 2.7, *p* < 0.01). As for diseases, only hyperlipidemia showed significant differences between men and women (427, 60.9 vs. 624, 71.3%, *p* < 0.01). As for OD, carotid plaque had the highest morbidity (68.0% overall). Besides, female participants had a higher rate of LVDD but a lower rate of intima-media thickness (IMT).

### Follow-Up Data

During the average 571 ± 135 days of follow-up, a total of 90 MACEs occurred without statistical differences between men and women (36, 5.1% vs. 54 6.2%, *p* = 0.38). As for the specific kind of MACE, more non-fatal myocardial infarction (MI) occurred in male participants (10, 1.4% vs. 3, 0.3%, *p* = 0.02) while more non-fatal stroke occurred in female participants (22, 3.1% vs. 46, 5.3%, *p* = 0.04). At the end of follow-up, nine cases of CVD death were observed, four for men (0.6%) and five for women (0.6%).

### Association Between Risk Factors and Major Adverse Cardiovascular Events

Univariable Cox regression was applied to all risk factors (Table 2). Some factors such as age (HR: 1.07, 95% CI: 1.04–1.11), eGFR (HR: 0.98, 95% CI: 0.97–0.99), DM (HR: 1.99, 95% CI: 1.189–2.719), stroke history (HR: 1.98, 95% CI: 1.27–3.08), and CHD family history (aHR: 1.82, 95% CI: 1.20–2.75) showed significant importance in predicting MACEs. In contrast, hypertension and hyperlipidemia were insufficient to predict MACEs. Among all anthropometric characters, larger MUAC was found as a protective factor for the MACE.

**TABLE 2:** Univariate Cox regression of risk factors for MACE.

Character	HR (95% CI)		*P*-value
**Cardiovascular risk factors**			
Gender (1 = male, 2 = female)	0.83	(0.55–1.27)	0.40
Age (years)	1.07	(1.04–1.11)	<0.01*
Family history of premature CVD (1 = yes, 2 = no)	1.15	(0.70–1.89)	0.59
Smoking, n (%)	1.29	(0.74–2.25)	0.36
TC (mmol/L)	0.97	(0.79–1.20)	0.78
TG (mmol/L)	1.02	(0.83–1.26)	0.85
HDLC (mmol/L)	0.73	(0.40–1.33)	0.30
LDLC (mmol/L)	0.98	(0.77–1.25)	0.87
SBP (mmHg)	0.99	(0.98–1.00)	0.21
DBP (mmHg)	0.98	(0.96–1.01)	0.15
eGFR (ml/min/1.73 m^2^)	0.98	(0.97–0.99)	<0.01*
**Anthropometric character**			
Height (cm)	0.97	(0.95–1.00)	0.04*
Weight (Kg)	0.99	(0.97–1.01)	0.16
Waist circumference (cm)	0.99	(0.97–1.02)	0.56
Hip circumference (cm)	0.99	(0.96–1.02)	0.60
MUAC (cm)	0.89	(0.82–0.96)	<0.01*
BMI (kg/m^2^)	1.00	(0.94–1.06)	0.97
WHR	0.55	(0.02–14.64)	0.72
Obesity (1 = yes, 2 = no)	0.75	(0.39–1.44)	0.38
Central obesity (1 = yes, 2 = no)	1.09	(0.70–1.69)	0.70
**Diseases**			
DM (1 = yes, 2 = no)	1.99	(1.30–3.07)	<0.01*
Hypertension (1 = yes, 2 = no)	1.15	(0.74–1.78)	0.54
Hyperlipidemia (1 = yes, 2 = no)	0.86	(0.56–1.32)	0.49
Stroke history (1 = yes, 2 = no)	1.98	(1.27–3.08)	<0.01*
CHD history (1 = yes, 2 = no)	1.82	(1.20–2.75)	0.01*

*Association of MACE with all risk factors was analyzed by univariate Cox regression.*

*P-values < 0.05 are considered significant.

*CVD, cardiovascular disease; TC, triglyceride; TG, total cholesterol; HDLC, high density lipoprotein cholesterol; LDLC, low density lipoprotein cholesterol; SBP, systolic blood pressure; DBP, diastolic blood pressure; eGFR, estimated glomerular filtration rate; MUAD, middle-up arm circumference; BMI, body mass index; WHR, waist-to-hip ratio; DM, diabetes mellitus; CHD, coronary heart disease.*

**FIGURE 1:**
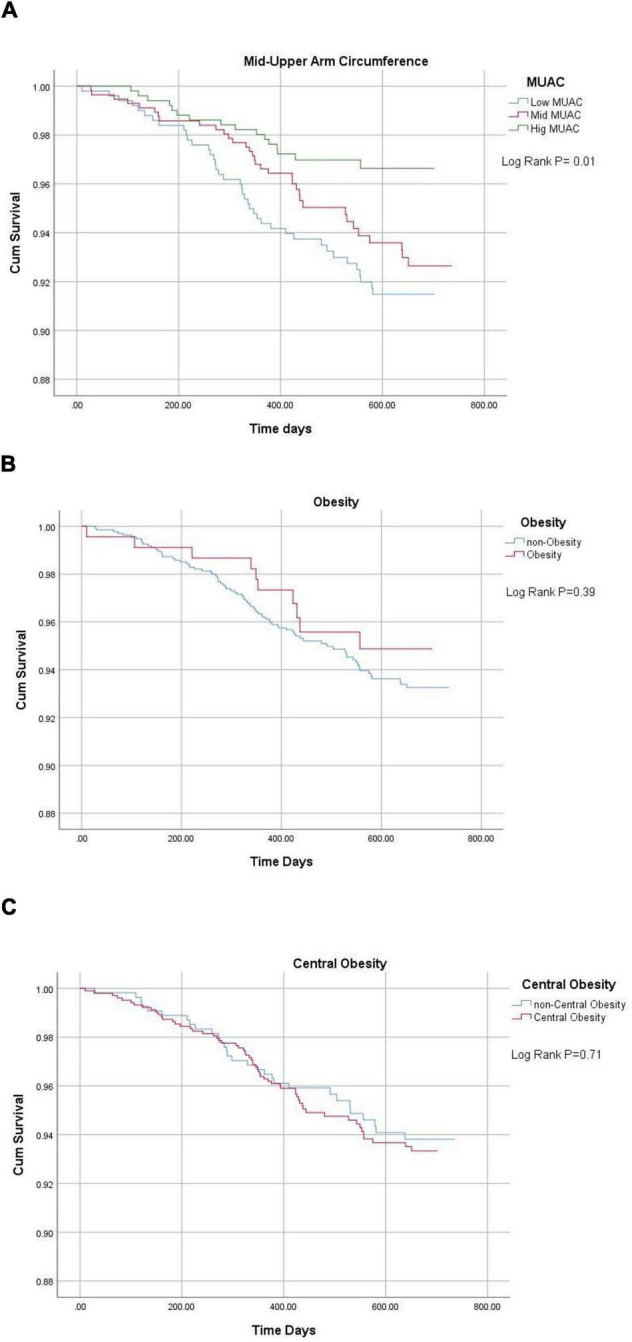
Kaplan-Meier plots of anthropometry factors for MACE. **(A)** Male and female participants were, respectively, divided into three groups according to the tertiles of MUAC level (low group: male MUAC ≤ 26.3 cm, female MUAC ≤ 25.9 cm; mid group: male < 26.3 ≤ MUAC ≤ 28.5 cm, female 25.0 < MUAC ≤ 28.0 cm; and high group: male MUAC > 28.5 cm, female > 28.0 cm). **(B)** Participants were divided into obesity (BM ≥ 28 kg/m^2^) and non-obesity. **(C)** Participants were divided into central obesity (WC ≥ 85 cm for male and ≥ 80 cm for female) and non-central obesity. MUAD, middle-up arm circumference.

The Kaplan-Meier survival curves are plotted in Figure 1, illustrating a similar result that a high MUAC group resulted in the lowest MACE risk than the other two groups (log-rank = 0.01).

### Association of Anthropometric Characters With Risk Factors and Organ Damage

All participants were divided into three groups and all risk factors were included in comparison through the rank sum test. As a result (Table 3), a high MUAC group had higher TC, TG, LDL-C, SBP, and DBP but lower HDL-C. The high MUAC group also had higher BMI, WC, and WHR.

**TABLE 3:** Risk factors by levels of MUAC.

Characteristic	MUAC low		MUAC mid		MUAC high		*P*-value
N	505		563		508		
**Cardiovascular risk factors**							
Age (years)	72.3	±6.0	72.0	±6.1	71.5	±5.9	0.08
Gender (male)	236	(46.7)	210	(37.3)	255	(50.2)	<0.01*
Family history of premature CVD, N (%)	98	(19.4)	122	(21.8)	101	(19.9)	0.60
Smoking, N (%) (male)	72	(30.5)	53	(25.1)	77	(30.2)	0.37
TC (mmol/L)	5.11	±0.99	5.29	±0.97	5.24	±1.07	0.02*
TG (mmol/L)	1.45	±0.96	1.58	±0.87	1.78	±0.94	<0.01*
HDLC (mmol/L)	1.44	±0.38	1.40	±0.37	1.29	±0.30	<0.01*
LDLC (mmol/L)	3.09	±0.86	3.26	±0.83	3.24	±0.85	<0.01*
SBP (mmHg)	129.4	±17.9	134.9	±17.3	139.5	±16.4	<0.01*
DBP (mmHg)	76.1	±8.1	79.1	±9.2	82.4	±9.1	<0.01*
eGFR (ml/min/1.73 m^2^)	82.7	±15.4	83.1	±14.9	82.9	±15.4	0.89
**Anthropometric character**							
Height (cm)	159.4	±8.0	159.0	±8.0	161.4	±8.6	<0.01*
Weight (Kg)	55.6	±8.4	61.2	±8.0	70.6	±9.5	<0.01*
Waist circumference (cm)	79.4	±7.7	85.5	±8.2	92.3	±8.7	<0.01*
Hip circumference (cm)	92.2	±5.7	96.9	±5.7	102.2	±6.8	<0.01*
BMI (kg/m^2^)	21.9	±2.7	24.2	±2.5	27.1	±3.1	<0.01*
WHR	0.86	±0.06	0.88	±0.06	0.90	±0.06	<0.01*
Obesity, n (%)	9	(1.8)	41	(7.3)	178	(25.0)	<0.01*
Central obesity, n (%)	182	(36.0)	399	(70.9)	451	(88.8)	<0.01*
**Diseases**							
DM, n (%)	101	(20.0)	119	(21.1)	127	(25.0)	0.13
Hypertension, n (%)	265	(52.5)	359	(63.8)	390	(76.8)	<0.01*
Hyperlipidemia, n (%)	285	(56.4)	395	(70.2)	371	(73.0)	<0.01*
Stroke history	98	(19.4)	112	(19.9)	105	(20.7)	0.88
CHD history	160	(31.7)	192	(34.1)	188	(37.0)	0.20

*Continuous data were presented as mean ± standard deviation and categorical variables by absolute numbers or percentage. Comparisons of continuous variables and categorical variables between different groups of MUAC were done by analysis of variance (ANOVA) and χ^2^ test with respectively.*

*P-values < 0.05 are considered significant.

*CVD, cardiovascular disease; TC, triglyceride; TG, total cholesterol; HDLC, high density lipoprotein cholesterol; LDLC, low density lipoprotein cholesterol; SBP, systolic blood pressure; DBP, diastolic blood pressure; eGFR, estimated glomerular filtration rate; MUAC, middle-up arm circumference; BMI, body mass index; WHR, waist-to-hip ratio; DM, diabetes mellitus; CHD, coronary heart disease.*

In univariable regression for OD (Table 4A), MUAC was profoundly associated with LVH and LVDD in both genders. Nevertheless, it had a poor correlation with vascular damage and renal damage. Considering anthropometric characters and other risk factors in the multivariate stepwise regression model (Table 4B), MUAC remained a good predictor for LVH in women and for LVDD in both genders. Other findings could also be safely drawn, for example, MUAC was a predictor of IMT > 0.9 mm in men and WHR was a predictor of renal damage.

**TABLE 4A:** Association of OD with anthropometric factors analyzed by univariate regression model.

Variables	LVH			LVDD			IMT > 0.9 mm			CF-PWV > 12 m/s			Caroid plaque			Renal damage		
	OR (95% CI)		*p*-value	OR (95% CI)		*p*-value	OR (95% CI)		*p*-value	OR (95% CI)		*p*-value	OR (95% CI)		*p*-value	OR (95% CI)		*p*-value
**Overall**																		
MUAC per sd	1.39	1.20–1.62	<0.01*	1.45	1.25–1.69	<0.01*	1.32	1.03–1.68	0.03*	1.05	0.90–1.21	0.57	1.07	0.96–1.18	0.21	1	0.82–1.17	0.79
BMI per sd	1.44	1.24–1.67	<0.01*	1.44	1.24–1.66	<0.01*	1.14	0.90–1.46	0.28	1.26	1.08–1.45	<0.01*	1.05	0.95–1.16	0.36	1.2	0.99–1.40	0.06
WC per sd	1.47	1.26–1.71	<0.01*	1.30	1.13–1.51	<0.01*	1.27	1.00–1.61	0.05	1.37	1.18–1.59	<0.01*	0.95	0.85–1.05	0.27	1.4	1.18–1.67	<0.01*
WHR per sd	1.20	1.04–1.37	0.01*	1.12	0.97–1.28	0.12	1.16	0.93–1.44	0.19	1.26	1.10–1.45	<0.01*	0.93	0.85–1.03	0.15	1.4	1.17–1.62	<0.01*
**Male**																		
MUAC per sd	1.31	1.02–1.68	0.03*	1.59	1.19–2.11	<0.01*	1.64	1.18–2.29	<0.01*	1	0.79–1.27	0.99	0.84	0.71–0.995	0.04*	0.9	0.71–1.22	0.59
BMI per sd	1.53	1.20–1.94	<0.01*	1.44	1.09–1.88	0.02*	1.21	0.87–1.67	0.26	1.24	0.97–1.57	0.08	0.85	0.82–1.01	0.06	1.1	0.86–1.49	0.39
WC per sd	1.47	1.17–1.87	<0.01*	1.35	1.04–1.76	0.03*	1.25	0.92–1.71	0.15	1.29	1.02–1.62	0.03*	0.88	0.75–1.03	0.11	1.3	0.995–1.68	0.05
WHR per sd	1.19	0.94–1.52	0.15	1.09	0.82–1.44	0.56	1.21	0.88–1.67	0.25	1.28	1.01–1.63	0.04*	0.94	0.80–1.11	0.45	1.3	1.02–1.76	0.04*
**Female**																		
MUAC per sd	1.47	1.21–1.78	<0.01*	1.48	1.24–1.77	<0.01*	0.92	0.60–1.38	0.7	1.09	0.89–1.32	0.41	0.96	0.84–1.11	0.59	1	0.80–1.28	0.93
BMI per sd	1.39	1.16–1.67	<0.01*	1.44	1.21–1.71	<0.01*	1.08	0.74–1.58	0.7	1.26	1.05–1.52	0.01*	1.02	0.89–1.17	0.79	1.2	0.97–1.52	0.09
WC per sd	1.55	1.27–1.91	<0.01*	1.47	1.22–1.78	<0.01*	1.09	0.72–1.64	0.68	1.49	1.22–1.83	<0.01*	1.10	0.95–1.28	0.2	1.5	1.20–1.97	<0.01*
WHR per sd	1.27	1.06–1.51	0.01*	1.3	1.09–1.53	<0.01*	0.89	0.60–1.33	0.56	1.31	1.09–1.57	<0.01*	1.05	0.91–1.20	0.5	1.4	1.15–1.79	<0.01*
MUAC per sd	1.31	1.02–1.68	0.03*	1.59	1.19–2.11	<0.01*	1.64	1.18–2.29	<0.01*	1	0.79–1.27	0.99	0.84	0.71–0.995	0.04*	0.9	0.71–1.22	0.59
BMI per sd	1.53	1.20–1.94	<0.01*	1.44	1.09–1.88	0.01*	1.21	0.87–1.67	0.26	1.24	0.97–1.57	0.08	0.85	0.82–1.01	0.06	1.1	0.86–1.49	0.39
WC per sd	1.47	1.17–1.87	<0.01*	1.35	1.04–1.76	0.03*	1.25	0.92–1.71	0.15	1.29	1.02–1.62	0.03*	0.88	0.75–1.03	0.11	1.3	0.995–1.68	0.05
WHR per sd	1.19	0.94–1.52	0.15	1.09	0.82–1.44	0.56	1.21	0.88–1.67	0.25	1.28	1.01–1.63	0.04*	0.94	0.80–1.11	0.45	1.3	1.02–1.76	0.04*

*Association of OD with MUAC, BMI, WC, and WHR was analyzed by univariate regression.*

*P-values < 0.05 are considered significant.

**TABLE 4B:** Association of OD with anthropometric factors analyzed by multivariate regression model.

Variables included in the model	LVH			LVDD			IMT > 0.9 mm			CF-PWV > 12 m/s			Caroid plaque			Renal damage		
	OR (95% CI)		*p*-value	OR (95% CI)		*p*-value	OR (95% CI)		*p*-value	OR (95% CI)		*p*-value	OR (95% CI)		*p*-value	OR (95% CI)		*p*-value
Overall																		
Age per sd	1.2	1.03–1.38	0.02*	1.25	1.08–1.45	<0.01*				2.02	1.74–2.34	<0.01*	1.44	1.28–1.61	<0.01*	2.67	2.23–3.21	<0.01*
Gender (male = 1, female = 2)	0.7	0.52–0.98	0.04*	0.41	0.29–0.57	<0.01*	2.06	1.24–3.43	0.01*									
MUAC per sd				1.45	1.24–1.70	<0.01*	1.29	1.01–1.66	0.045*									
BMI per sd																		
WC per sd	1.4	1.18–1.63	<0.01*															
WHR per sd																1.29	1.08–1.54	0.01*
DM (yes = 1, no = 0)				1.45	1.03–2.04	0.03*				2.42	1.70–3.35	<0.01*	1.50	1.14–1.97	<0.01*			
Hypertension (yes = 1, no = 0)	2.33	1.57–3.44	<0.01*	2.19	1.51–3.19	<0.01*				2.68	1.78–4.03	<0.01*						
Hyperlipidemia (yes = 1, no = 0)															1.97	1.27–3.07	0.01*	
Smoking (yes = 1, no = 0)																		
Male																		
Age per sd										2.03	1.61–2.55	0.001	1.33	1.12–1.58	0.001	2.90	2.19–3.84	0.001
MUAC per sd				1.45	1.08–1.95	0.02*	1.65	1.18–2.30	<0.01*									
BMI per sd	1.4	1.08–1.79	0.01*															
WC per sd																		
WHR per sd																		
DM (yes = 1, no = 0)										2.30	1.35–3.92	<0.01*				2.02	1.08–3.78	0.03
Hypertension (yes = 1, no = 0)	2.3	1.24–4.42	0.01*	3.09	1.42–6.72	<0.01*				2.49	1.32–4.70	0.01						
Hyperlipidemia (yes = 1, no = 0)															2.70	1.40–5.20	<0.01*	
Smoking (yes = 1, no = 0)																		
Female																		
Age per sd	1.3	1.10–1.61	<0.01*	1.32	1.11–1.57	<0.01*				2.02	1.66–2.46	<0.01*	1.50	1.29–1.76	<0.01*	2.48	1.96–3.15	<0.01*
MUAC per sd	1.4	1.16–1.73	<0.01*	1.44	1.20–1.73	<0.01*												
BMI per sd																		
WC per sd																		
WHR per sd																1.30	1.03–1.63	0.03
DM (yes = 1, no = 0)				1.57	1.05–2.35	0.03*				2.42	1.56–3.76	<0.01*	1.53	1.06–2.22	0.02			
Hypertension (yes = 1, no = 0)	2.30	1.40–3.87	<0.01*	1.97	1.28–3.04	<0.01*				2.84	1.67–4.85	<0.01*						
Hyperlipidemia (yes = 1, no = 0)																		
Smoking (yes = 1, no = 0)																		

*Association of OD with MUAC, BMI, waist circumference, and WHR was analyzed by multivariate stepwise regression when MUAC, BMI, waist circumference, and WHR were simultaneously put into the model.*

*P-values < 0.05 are considered significant.

*OD, organ damage; MUAC, middle-up arm circumference; BMI, body mass index; WC, waist circumference; WHR, waist-to-hip ratio; LVH, Left ventricular hypertrophy; LVDD, left ventricular diastolic dysfunction; IMT, carotid intima-media thickness; CF-PWV, carotid-femoral pulse wave velocity.*

### Association of Different Anthropometric Characters With the Major Adverse Cardiovascular Events

Table 5 shows the results from both univariate and multivariate Cox regression analyses of anthropometric characters, including MUAC, BMI, WHR, and WC for MACE. The univariable cox regression of all participants revealed that only MUAC had a predictive and protective association with the MACE (HR: 0.73, 95% CI: 0.59–0.89). After being adjusted for age, gender, hypertension, DM, hyperlipidemia, and smoking, MUAC remained protective for the MACE (aHR: 0.73, 95% CI: 0.59–0.91), while BMI, WHR, and WC showed no remarkable impact. The further model included eGFR, stroke history, and CHD family history into adjustment, getting the same result that only MUAC could play a prognostic role in the MACE (aHR: 0.73, 95% CI: 0.59–0.91). In the subgroup analysis, the result of men remained the same, but a marginally significant association in women was observed.

**TABLE 5:** Univariate and multivariate Cox regression models for MACE.

	MUAC per SD			BMI per SD			Waist circumference per SD			WHR per SD		
	HR	95% CI	*p*-value	HR	95% CI	*p*-value	HR	95% CI	*p*-value	HR	95% CI	*p*-value
**Overall**												
Model 1	0.73	0.59–0.89	<0.01*	0.996	0.81–1.23	0.97	0.94	0.76–1.16	0.55	0.96	0.79–1.17	0.71
Model 2	0.73	0.59–0.91	<0.01*	0.96	0.77–1.19	0.71	0.85	0.68–1.06	0.14	0.87	0.70–1.08	0.20
Model 3	0.73	0.59–0.91	<0.01*	0.94	0.75–1.17	0.57	0.84	0.67–1.05	0.12	0.87	0.70–1.09	0.22
**Male**												
Model 1	0.68	0.50–0.94	0.02*	0.91	0.64–1.30	0.62	0.91	0.66–1.26	0.58	0.96	0.69–1.35	0.81
Model 2	0.67	0.47–0.94	0.02*	0.92	0.64–1.33	0.67	0.88	0.62–1.24	0.46	0.91	0.63–1.30	0.62
Model 3	0.69	0.49–0.97	0.03*	0.92	0.63–1.33	0.65	0.88	0.63–1.25	0.48	0.92	0.64–1.31	0.64
**Female**												
Model 1	0.76	0.58–1.00	0.05	0.74	0.81–1.35	0.74	0.99	0.75–1.30	0.92	1.00	0.78–1.29	0.998
Model 2	0.77	0.59–1.02	0.07	0.97	0.74–1.27	0.85	0.82	0.61–1.11	0.20	0.83	0.62–1.11	0.21
Model 3	0.77	0.59–1.01	0.06	0.95	0.73–1.25	0.73	0.80	0.60–1.08	0.15	0.83	0.61–1.12	0.22

*Model 1: Univariate Cox regression.*

*Model 2: Adjusted by age, gender, hypertension, diabetes mellitus, hyperlipidemia, and smoking (only in the model of male participants and overall).*

*Model 3: Adjusted by age, hypertension, diabetes mellitus, hyperlipidemia, estimated glomerular filtration rate, stroke history, coronary heart disease history, and smoking (in the model of male participants and overall).*

*Significance of p < 0.05.

**FIGURE 2:**
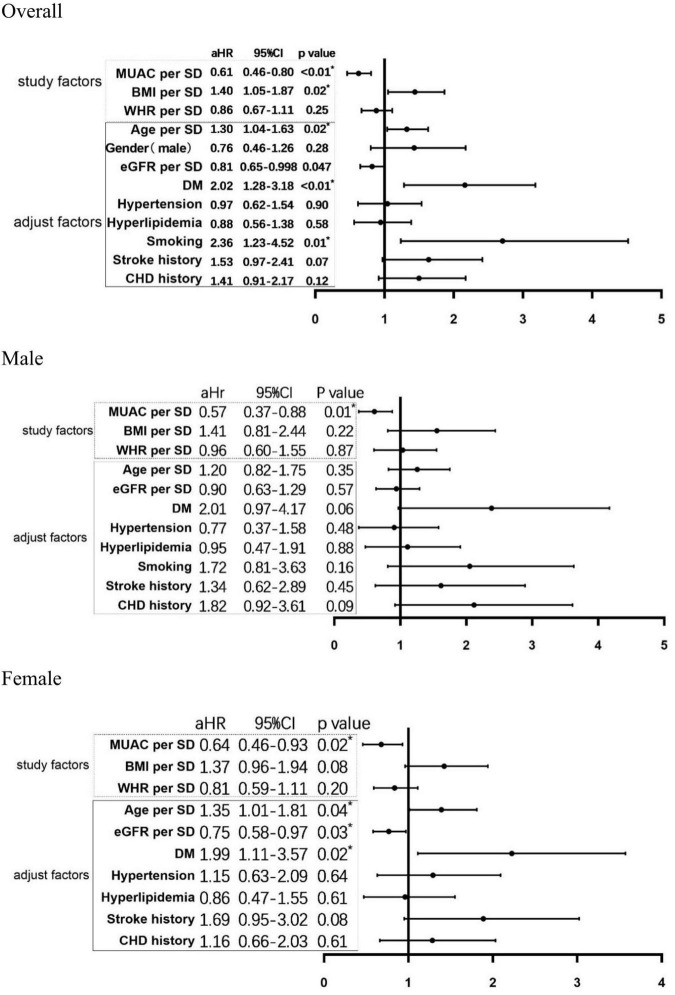
Multivariate Cox regression of risk factors for MACE. MUAC, middle-upper arm circumference; BMI, body mass index; eGFR, estimated glomerular filtration rate; DM, diabetes mellitus; CHD, coronary heart disease. *P-value < 0.05 were considered significant.

The final model of cox regression involved BMI, MUAC, WHR, and all other factors into adjustment (Figure 2). As a result, MUAC and eGFR were protective factors for the MACE, while age, BMI, DM, and smoking were associated with the increasing risk of the MACE. In women, the analysis revealed a similar result, except for the BMI that indicated no significant importance. The subgroup analysis of men showed MUAC to be the only predictor of lower MACE risk.

**FIGURE 3:**
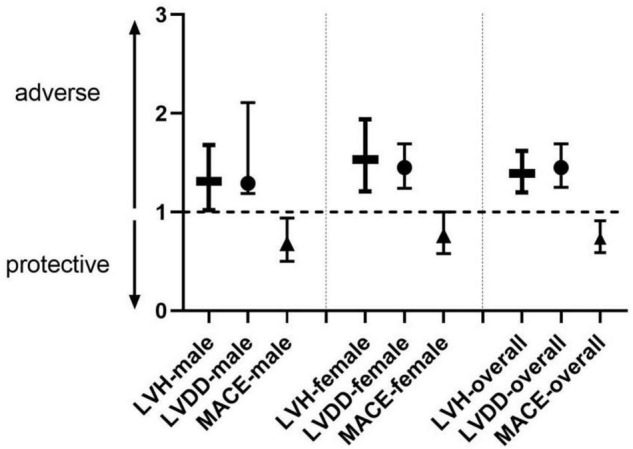
Association of LVH, LVDD with MUAC analyzed by univariate regression in male, female, and overall. aHR of MUAC for MACE analysis by multivariate Cox regression adjusted by age, BMI < WHR, hypertension, diabetes mellitus, hyperlipidemia, estimated glomerular filtration rate, stroke history, coronary heart disease history, and smoking (only in the model of male and overall). MUAC, middle-up arm circumference; LVH, left ventricular hypertrophy; LVDD, left ventricular diastolic dysfunction; MACE, major adverse cardiovascular event.

Finally, the paradoxical association with MUAC was analyzed with LVH, LVDD, and MACE by univariate regression and is shown in Figure 3.

## Discussion

The North Shanghai Study is an ongoing prospective cohort study. The primary findings of our study were as follows: (1) MUAC was an independent predictor of reduction of the MACE after adjustment by conventional CVD risk factors; (2) Higher MUAC was strongly associated with more risk factors and cardio damage.

MUAC has been utilized as a neurological marker to evaluate the nutritional status of children and adolescents (17–19). Further studies revealed its value in prognostic prediction for patients suffering from CVD (20). A national study of the United States with a total of 11,958 study cohorts revealed an inversive association between MUAC and all-cause mortality in US non-obese population (11). High MUAC was confirmed as a protective factor for all-cause and cause-specific mortality globally (21, 22).

The mechanism behind the association between high MUAC and a favorable prognosis in general population or patients with specific diseases remains unknown. As an anthropometry character, MUAC is a comprehensive description about fat-free mass and peripheral adipose. Body weight and BMI have a poor capability of identifying the proportion and absolute mass of the body component.

A previous study found that high MUAC was associated with a series of cardiovascular risk factors such as hypertension, hyperlipidemia, and DM (11). In our study, participants in the high MUAC group were associated with higher weight, BMI, WC, and WHR, which indicated more obesity and central obesity. However, they shared similar DM morbidity with the other two groups. It has been widely accepted that obesity is a major risk factor for DM (23). DM rather than hypertension or hyperlipidemia increased the MACE risk in our study. This might be caused by a different order of adipose tissue accumulation. In participants with high MUAC, their fat is preferentially stored in peripheral depots which is associated with more favorable effects on arterial stiffness and cardiovascular risk than on the trunk (10).

As another risk factor of the MACE, eGFR also showed no statistical difference in patients with three levels of MUAC. In contrast, WHR was found to be a predictor of renal damage. A previous study has already proved that the association of higher WHR and lower eGFR is intermediated by an altered renal hemodynamic index associated with body fat distribution and independent of the BMI (24).

In the meantime, we analyzed the effects of different anthropometry characters on OD. MUAC, BMI, and WC were all profoundly associated with LHV and LVDD, while WHR showed no correlation. Obesity had an adverse impact on the hemodynamic change and cardio structure which could often manifest as LVH and LVDD.

The initial development of LVH is a compensatory process which allows the heart to meet the increased cardio output demand with abnormal pressure or volume load (25). LVH is known as the most common pathological inducement and propulsion of LVDD (26, 27). Obesity increases total blood volume, stroke volume, and cardiac output which lead to more cardiovascular risk and left ventricular (LV) changes such as LV dilation and LVH (7). Physiological LVH can automatically reverse while pathological LVH is linked to higher risk of cardiovascular diseases and mortality (28). The LV mass is more strongly related to fat-free-mass than fat-mass, WHR, or BMI (29). BMI is defined as the height-normalized body weight which contains both fat-free-mass and fat-mass. Higher fat-free-mass can be observed in individuals with higher body weight as an adaptation. In a meta-analysis including 2.9 million individuals, overweight (BMI of 25 to <30) and grade 1 obesity (BMI of 30 to <35) groups showed better survival than the normal-BMI group (6). Patients with CDH with severe obesity (BMI ≥ 35) have favorable short-term outcomes but higher long-term mortality (30). In our study, the BMI of the participants was 27.1 ± 3.1 and the proportion of obesity was 25% in the high MUAC group, which means that it is not a severe obese population. In such a circumstance, the explanation of the paradoxical phenomenon that higher MUAC is linked to a higher risk of both LVH and LVDD but a lower risk of the MACE is that the increase of cardiac workload in overweight and mild obese population is still compensable and higher MUAC reflects better adaptation capability. However, this hypothesis needs further evidence.

As a reliable measurement of skeletal muscle, MUAC has been confirmed to be associated with not only muscle mass but also muscle strength (31, 32). Low muscle strength is a strong and independent predictor of mortality in older adults, and this association cannot be explained by low muscle mass (33). Skeletal muscle has been recognized as a paracrine and endocrine organ (34). For example, follistatin like 1 (FSTL-1), a glycoprotein secreted by skeletal muscle, can be inducted rapidly by short time exercise (35). In animal models, FSTL-1 can attenuate neointimal formation, promote endothelial cell function, and improve revascularization (36, 37).

A recent study supposed that MUAC was a predictor of central obesity (38). Central obesity identified by either WC or WHR is a risk factor for CVD (15, 39, 40). In our study, neither WHR nor WC could predict the MACE. The controversies may be caused by a relative low level of BMI (24.4 ± 3.5) and a low rate of obesity (228/1,576, 14.5%). The study conducted by Cho et al. highlighted that visceral obesity, rather than central obesity, was independently associated with structural and functional cardiac remodeling (41).

In our study, BMI showed its adverse impact on the MACE adjusted by MUAC, suggesting that there was a complementary effect between BMI and MUAC.

With the aging society problem getting worse every year in both China and developed countries, the primary health care plays an increasingly important role. Compared with blood test and body component analysis conducted by X-ray or MRI, MUAC is easy acquired, inexpensive, and non-invasive. The equipment request is only a regular tape-measure and non-professional social workers can be a competent for this study. Spinal deformities and body fluid changes can interfere with the measure of body height and body weight. Another body measure character, i.e., calf circumference, was not included in our study for the prevalence of fluid retention among elderly population while MUAC does not have these concerns.

In summary, we suggest that MUAC could be considered an easily acquired and beneficial risk factor reflecting muscle mass and peripheral adipose.

### Limitation

The study participants are typical community dwelling urban elderly citizens. All participants were over 65 years of age (average 72.0 ± 6.0 years) when enrolled. Most participants had been of after-retirement status and out of manual labor. They only involved in household duties and low-intensity exercises such as walking and square dancing. Our previous study (42) showed a negative association of weekly walking activity with hypertensive mediated vascular organ damage but not cardiac or renal damage. Our study is an observational study which makes it impossible to tell if the higher MUAC obtained by intentional upper limb exercise has the same protective effect. Hence, determining whether our results can be applied to suburban and country residents who still engage in manual labor or younger population whose physical activities are more intense and frequent needs further study.

Another limitation is that we only measured the left arm. The proportion of left-handed population is very low in China especially among elderly citizens. A survey of more than 20,000 mainland Chinese students and professionals in the 1980s reported that only 0.23% were left-handed (43). However, in other regions with a non-negligible left-handed rate, the measure of both sides should be considered.

## Conclusion

Despite being associated with more risk factors and cardio damage, MUAC is an independent protective predictor that has the capability of protectively predicting the MACE over BMI, WC, and WHR, in the Chinese elderly cohort.

## Data Availability Statement

The raw data supporting the conclusions of this article will be made available by the authors, without undue reservation.

## Ethics Statement

The studies involving human participants were reviewed and approved by the Ethics Committee of Shanghai Tenth People’s Hospital. The patients/participants provided their written informed consent to participate in this study.

## Author Contributions

YXZ, JZ, ZR, WM, JDT, SZ, CC, JX, JMT, and RM performed the measurements and acquired the primary data for the study. YZ and YX formulated the methods and designed the study protocol. YXZ and WM measured the cf_PWV and anthropometric indices. JX and YZ examined the echocardiography for each participant. YXZ completed the data analysis and drafted the manuscript. YZ assisted with the language review. All authors contributed to revisions and approved the final version of the manuscript.

## Conflict of Interest

The authors declare that the research was conducted in the absence of any commercial or financial relationships that could be construed as a potential conflict of interest.

## Publisher’s Note

All claims expressed in this article are solely those of the authors and do not necessarily represent those of their affiliated organizations, or those of the publisher, the editors and the reviewers. Any product that may be evaluated in this article, or claim that may be made by its manufacturer, is not guaranteed or endorsed by the publisher.
